# Tracking elimination of HIV transmission in men who have sex with men in England: a modelling study

**DOI:** 10.1016/S2352-3018(21)00044-8

**Published:** 2021-06-09

**Authors:** Francesco Brizzi, Paul J Birrell, Peter Kirwan, Dana Ogaz, Alison E Brown, Valerie C Delpech, O Noel Gill, Daniela De Angelis

**Affiliations:** a Medical Research Council Biostatistics Unit, University of Cambridge, Cambridge, UK; b National Infection Service, Public Health England, Colindale, UK

## Abstract

**Background:**

To manage the HIV epidemic among men who have sex with men (MSM) in England, treatment as prevention strategies based on test and treat were strengthened between 2011 and 2015, and supplemented from 2015 by scale-up of pre-exposure prophylaxis (PrEP). We examined the effect of these interventions on HIV incidence and investigated whether internationally agreed targets for HIV control and elimination of HIV transmission by 2030 might be within reach among MSM in England.

**Methods:**

We used a novel, age-stratified, CD4-staged Bayesian back-calculation model to estimate HIV incidence and undiagnosed infections among adult MSM (age ≥15 years) during the 10-year period between 2009 and 2018. The model used data on HIV and AIDS diagnoses routinely collected via the national HIV and AIDS Reporting System in England, and knowledge on the progression of HIV through CD4-defined disease stages. Estimated incidence trends were extrapolated, assuming a constant MSM population from 2018 onwards, to quantify the likelihood of achieving elimination of HIV transmission, defined as less than one newly aquired infection per 10 000 MSM per year, by 2030.

**Findings:**

The peak in HIV incidence in MSM in England was estimated with 80% certainty to have occurred in 2012 or 2013, at least 1 year before the observed peak in new diagnoses in 2014. Results indicated a steep decrease in the annual number of new infections among MSM, from 2770 (95% credible interval 2490–3040) in 2013 to 1740 (1500–2010) in 2015, followed by a steadier decrease from 2016, down to 854 (441–1540) infections in 2018. A decline in new infections was consistently estimated in all age groups, and was particularly marked in MSM aged 25–34 years, and slowest in those aged 45 years or older. Similar trends were estimated in the number of undiagnosed infections, with the greatest decrease after 2013 in the 25–34 years age group. Under extrapolation assumptions, we calculated a 40% probability of achieving the defined target elimination threshold by 2030.

**Interpretation:**

The sharp decrease in HIV incidence, estimated to have begun before the scale up of PrEP, indicates the success of strengthening treatment as prevention measures among MSM in England. To achieve the 2030 elimination threshold, targeted policies might be required to reach those aged 45 years or older, in whom incidence is decreasing at the slowest rate.

**Funding:**

UK Medical Research Council, UK National Institute of Health Research Health Protection Unit in Behavioural Science and Evaluation, and Public Health England.

## Introduction

In 2015, the UN member states adopted the Sustainable Development Goals, including the Goal 3 target to end the AIDS epidemic by 2030. Adoption of this target followed the 2014 UNAIDS fast-track strategy for elimination of HIV transmission that set the 90-90-90 targets (90% of people living with HIV being diagnosed; 90% of diagnosed individuals receiving antiretroviral therapy [ART]; and 90% of people on ART being virally suppressed by 2020).^1^ Among men who have sex with men (MSM) in England, regular monitoring of new HIV diagnoses, HIV care, and prevalence estimations have shown a steady decrease in new HIV diagnoses since 2015, and showed achievement of the 90-90-90 target by the end of 2016.^2^ A new goal in England is to reach elimination of HIV as a public health problem by 2030, including in MSM.^3^

Although trends in new HIV diagnoses are a valuable metric, new diagnoses during any period do not equate to the new infections in that period. Diagnoses are a dynamic mixture of long-standing and recent HIV infections, resulting from the complex interplay between transmission, infection progression, and testing uptake. Only by modelling these processes can researchers distinguish the contributions of changes in testing intensity and transmission to the observed HIV diagnoses, and thus estimate the underlying number of new infections. The CD4-staged back-calculation approach reconstructs HIV incidence and estimates time-varying diagnosis rates by use of information on new HIV and AIDS diagnoses, CD4 cell counts around diagnosis, and the natural course of HIV infection.^4^ In addition, the method estimates the number of undiagnosed infections over time and trends in the time interval from infection to diagnosis.^5^

**Research in context**

**Evidence before this study**

The UN Sustainable Development Goals (SDGs) include the target to end HIV as a public health threat by 2030. To achieve this target, HIV elimination strategies in developed countries have included increased testing, and treatment as prevention, with pre-exposure prophylaxis (PrEP) now considered the best strategy to prevent HIV transmission among men who have sex with men (MSM). In England in 2013, a back-calculation analysis of various HIV surveillance data found that a decade of test-and-treat strategies had not been able to detectably reduce HIV incidence in MSM by the end of 2010. This work led to the advocacy to rethink HIV prevention strategies. Since 2011, test-and-treat measures have been intensified, and PrEP uptake progressively increased among MSM from 2016. A decrease in the number of new diagnoses among this group has been evident since 2015. However, it is unclear whether this decrease was due to reduced HIV incidence, and, if so, when a reduction in incidence began and what might explain it. We searched studies on PubMed related to both estimating HIV incidence in MSM and HIV elimination prospects. We undertook two searches, using a 2010–20 data range and no language restrictions. For the first search we used the keywords (estimating or estimation) AND (hiv or HIV) AND (incidence) AND (men who have sex with men OR msm OR MSM), yielding 1648 results; for the second search we used the keywords (hiv or HIV) AND (elimination) AND (public health), yielding 2197 results. We further checked for related studies by research groups linked to public health bodies in resource-rich countries, such as Australia, Canada, Denmark, the Netherlands, and the USA. We found no work comparable to our methodology that was being implemented as a component of routine surveillance.

**Added value of this study**

This study presents results from a novel, age-stratified back-calculation based on routinely collated HIV surveillance data to the end of 2018. Our method allowed estimation of age-specific trends in HIV incidence, undiagnosed prevalence, and time to diagnosis, and facilitated a principled incidence extrapolation to 2030. We estimated that the peak in new HIV infections occurred in 2012 or 2013, followed by a sustained decline to 2018, with this decrease particularly marked in young MSM. Through extrapolation, we evaluated the likelihood of England reaching the UN SDG elimination target by 2030 (in England, a target of less than one newly acquired infection per 10 000 at risk per year), and identified relevant age-specific targeting of further prevention efforts (ie, to MSM aged ≥45 years) to increase this likelihood. This work could not formally attribute cause or quantify the contributions of different components of combined HIV prevention to decreasing HIV incidence. However, our results show that the decrease in incidence began before the widespread roll-out of PrEP. Therefore, testing and treatment measures might have had an effective role in decreasing HIV in MSM in a resource-rich country against a backdrop of increasing incidence of sexually transmitted infections.

**Implications of all the available evidence**

In England, the amplified test-and-treat strategies since 2011 have been associated with a decrease in new HIV infections among MSM, ahead of the scale-up of PrEP. This conclusion can only be drawn from the estimated trends in HIV incidence, which show a decrease in transmission before the observed decrease in number of HIV diagnoses. Additionally, given a clear definition of HIV elimination in MSM, we have proposed a method for forecasting and quantifying the prospect of eliminating HIV transmission by 2030. Our study shows that elimination of HIV transmission, as defined by the UN SDG 2030 target, is a feasible aim in England, particularly as the full effect of widening access to PrEP is yet to be identified and might consolidate the estimated decrease in HIV incidence. Vital components of reaching the 2030 target will be ensuring that an increased number of MSM test more frequently, and that high rates of treatment access are maintained in the coming years.

For MSM in England during 2001–10, despite a large rise in HIV testing, increased retention in care, and improvements in the proportion of people living with HIV who were receiving ART, a back-calculation analysis showed a persistently high HIV incidence of around 2500 new infections annually.^6^ Other studies that used stochastic simulations estimated that HIV incidence in MSM increased during 2000–10^7^ and 2000–13.^8^ During these years in developed countries there was a resurgence in condomless sex (in England, from 43% to 52% between 2000 and 2013^9^), increases in sexualised drug use, and a rise in the number of sexual partnerships facilitated by greater online connectivity;^9^, ^10^, ^11^ thus, it seems probable that increased access to testing and treatment might have limited HIV transmission to some extent. Nevertheless, the plateau in HIV incidence was described as sobering, and led to doubts that testing and treatment efforts could substantially reduce HIV transmission in MSM.^12^

Since 2011, the biomedical components of combination HIV prevention for MSM have evolved in England. Initially, treatment as prevention had been advocated after mathematical modelling showed the effect of a universal test-and-treat policy on HIV incidence;^13^ ecological studies supported an inverse relationship between ART coverage and new HIV diagnoses;^14^ and the HIV Prevention Trials Network 052 trial showed early ART initiation could reduce sexual transmission in serodiscordant heterosexual couples.^15^ An HIV test every 3 months (rather than every 6 months) for the most at-risk MSM was recommended in 2012,^16^ and new guidelines were issued to offer ART to those with a CD4 count of less than 350 cells per μL,^17^ followed in 2013 by a position statement recommending that health-care professionals discuss use of ART to reduce risk of transmission with all people living with HIV.^18^ In 2015, immediate ART initiation was recommended for all people newly diagnosed with HIV infection.^19^, ^20^

In addition, following evidence of pre-exposure prophylaxis (PrEP) efficacy in MSM when taken consistently,^21^ the PROUD trial was initiated in England,^22^ and MSM at high risk of aquiring HIV were introduced to PrEP as part of the trial in 2012. Around 25 MSM were taking PrEP by the end of 2012, 250 by the end of 2013, and 500 (ie, every trial participant) by the end of 2014.^22^ In autumn, 2015, internet sites were established to facilitate self-purchase of PrEP from abroad. An online survey of MSM indicated that PrEP usage quadrupled during 2016, with an estimated 3000 MSM taking PrEP by the end of the year.^23^ Following the start of a large-scale PrEP implementation trial in October, 2017, the number of MSM taking PrEP was estimated to have increased progressively to around 5000 by the end of 2017 and 15 000 by the end of 2018.^24^

An incidence threshold below which HIV transmission might be considered eliminated in MSM in England has been defined as one new HIV infection per 10 000 MSM per year,^25^ equivalent to fewer than 50 new infections acquired annually in England. The likelihood of achieving this low incidence will depend on the lessons learned from combination prevention strategies in the past 10 years, which might differ across different MSM age groups. Crucially, HIV progression depends on age at infection,^26^ time from diagnosis to treatment varies between age groups,^27^ and trends in new HIV diagnoses and bacterial sexually transmitted infections (STIs) observed since 2010 also differ by age.^28^, ^29^ These heterogeneities could reveal a differential effect of combination prevention across age groups.

In this paper we have reconstructed the overall and age-specific HIV incidence trends for 2009–18 in MSM in England using a novel, age-stratified, CD4-staged back-calculation model.^30^ We investigated whether the incidence trends mirrored changes in the biomedical components of combination prevention during three key periods: early treatment as prevention (before 2011), pre-PrEP (2011–15), and post-PrEP (2016–18). In addition, we estimated the timing of the peak in incidence, and examined whether incidence changes were consistent across age groups. Furthermore, assuming remaining gaps in combination prevention are successfully addressed and, as a consequence, current incidence trends are maintained, we estimated the likelihood that the Sustainable Development Goal for eliminating HIV transmission might be reached by 2030.

## Methods

### Overview

To provide context on the evolution of the HIV epidemic among adult MSM (≥15 years) in England, routinely collected surveillance data are first presented. Subsequently, the novel, age-stratified, CD4-staged back-calculation approach was used to estimate age-specific HIV incidence, undiagnosed infections, and mean time to diagnosis in adult MSM. Incidence estimates were then extrapolated to predict the probability of achieving the HIV transmission elimination target by 2030.

### Data sources

Information on quarterly numbers of new HIV and AIDS diagnoses in MSM was obtained, together with age at diagnosis, linked data on CD4 cell counts within 3 months of diagnosis, and time of ART initiation, from the national HIV and AIDS Reporting System (HARS) of Public Health England. HARS receives reports from all National Health Service laboratories, hospitals, and clinics on a quarterly basis, with stringent data quality checks and ongoing follow-up of ART initiation and incomplete or inconsistent information. Since 2000, linked CD4 cell counts were available for 75–85% of newly diagnosed MSM and completeness of data on HIV exposure route exceeded 90% in all years excluding 2019–20 (ongoing follow-up). HARS reporting is linked to HIV service commissioning, and therefore the reporting delay is low. We used HARS data on adult MSM first diagnosed in England between 1995 and 2018, excluding individuals born abroad or previously diagnosed abroad. An AIDS diagnosis was an HIV diagnosis followed by AIDS-defining symptoms within 3 months, and time to treatment as the time from initial diagnosis to initiation of ART. All references for these data sources are given in the appendix (p 3).

We further extracted data on annual numbers of MSM on PrEP.^22^, ^24^ Additionally, annual numbers of HIV tests, including repeat tests, and diagnoses of gonorrhoea and syphilis (2010–18) among MSM were obtained from the Genitourinary Medicine Clinic Activity Dataset (GUMCAD) STI surveillance system. GUMCAD is a comprehensive, pseudoanonymised dataset of all individual-level STI services and diagnoses made at specialist sexual health clinics in England.^11^ Each pseudoanonymised record contains a clinic identifier and local patient number, allowing data from the same individual attending the same clinic to be linked longitudinally.

### Back-calculation analysis

The quarterly HIV and AIDS diagnoses and CD4 data were analysed with the novel, age-stratified, CD4-staged back-calculation model, an extension of the CD4-staged back-calculation model previously used to understand the HIV epidemic in England.^4^, ^5^ Briefly, the model describes HIV infection and diagnosis in the MSM population as progression through compartments: after infection, individuals proceed through decreasing CD4 cell count strata, and either remain undiagnosed or move into a diagnosed compartment. Disease progression depends on age at infection, and the probability of being diagnosed depends on CD4 cell count, age, and calendar time.

This back-calculation model allowed age-specific estimation of the quarterly number of new HIV infections, the quarterly HIV diagnosis probabilities and, indirectly, mean time to diagnosis and the quarterly number of undiagnosed infections among MSM. Quarterly estimates were summed to give yearly estimates. Age groups were based on those used for routine HIV surveillance up to age 44 years (15–24 years, 25–34 years, 35–44 years, and ≥45 years). More detailed information on the model and the assumptions made are provided in Brizzi et al^30^ and the appendix (pp 1–3).

### Incidence estimation and extrapolation

The annual number of new HIV infections was estimated with results presented for 2009–18, encompassing the three key phases of combination prevention in England: early treatment as prevention (before 2011), pre-PrEP (2011–15), and post-PrEP (2016–18). Annual incidence estimates were obtained by dividing the estimated number of new infections by the size of the HIV-negative MSM population, provided by complementary multiparameter evidence synthesis (MPES) work on HIV burden in England.^31^ To predict incidence after 2018 up to 2030, the estimated age–calendar time profile for the number of new infections was extrapolated (appendix p 2). The MSM population size was assumed to remain constant from 2018 onwards.

### Role of the funding source

The funders of the study had no role in study design, data collection, data analysis, data interpretation, or writing of the report.

## Results

**Figure 1:**
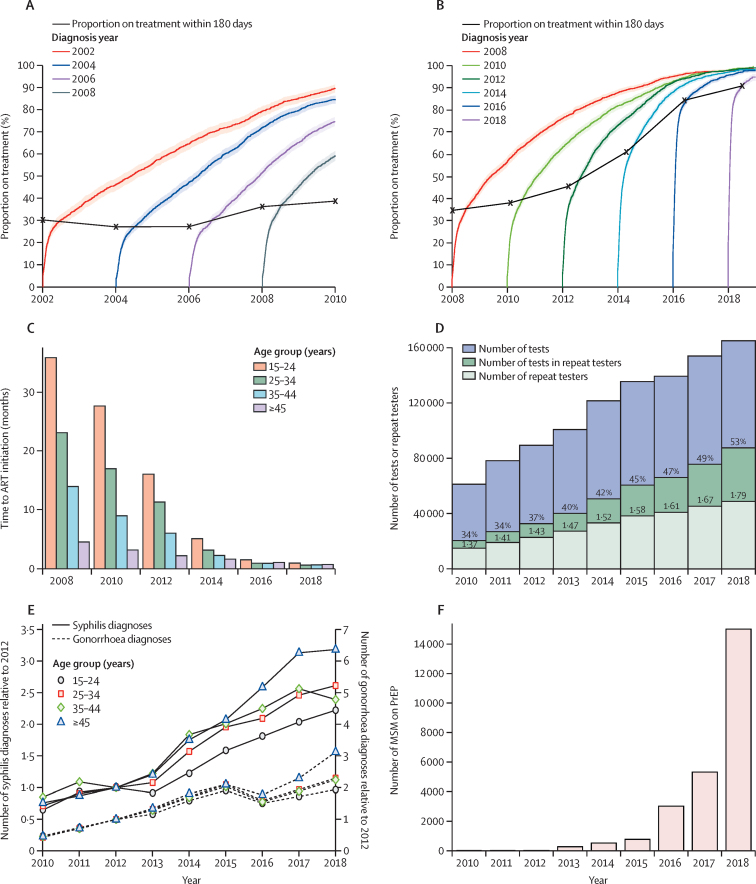
Trends in indicators of HIV prevention initiatives and risk exposure Cumulative distributions for the time to ART initiation for MSM diagnosed with HIV in 2002, 2004, 2006, and 2008 (A), and in 2008, 2010, 2012, 2014, 2016, and 2018 (B), highlighting the difference pre-2010 and post-2010. Shading around each line represents the 95% CIs for the proportions on treatment. (C) Median time to ART initiation after diagnosis by age group in 2008–18. (D) Annual numbers of HIV tests in MSM in sexually transmitted infection clinics, the most common setting for HIV tests in MSM, for 2010–18. Numbers above the light green bars are the mean number of tests taken in a year by a repeat tester; numbers above the dark green bars are the proportion of testers classified as repeat testers. (E) Annual number of syphilis diagnoses (left axis) and gonorrhoea diagnoses in all MSM (right axis) by age group, relative to the 2012 number of diagnoses in that age group. (F) Scale-up in the number of MSM on PrEP since 2010. ART=antiretroviral therapy. MSM=men who have sex with men. PrEP=pre-exposure prophylaxis.

Changes in the distribution of time to treatment initiation during the periods 2002–10 (figure 1A) and 2008–18 (figure 1B) show that a markedly greater improvement in treatment access was achieved after 2010 than in earlier years, with the cumulative distributions of the time to treatment rapidly increasing for HIV diagnoses in 2016 and 2018, indicating near immediate initiation of ART. The proportion of MSM on ART within 180 days of HIV diagnosis (a metric used by Public Health England to monitor the rapid uptake of ART among individuals newly diagnosed with HIV) increased from 34·7% in 2008 to 84·5% in 2016, and 91·0% in 2018. The observed improvement was mostly attributable to faster access to treatment in MSM aged 15–24 years (median time of 35·9 months in 2008, decreasing to 0·92 months in 2018) and those aged 25–34 years (23·1 months, decreasing to 0·59 months; figure 1C). MSM aged 45 years or older already had a short waiting time to treatment (4·53 months decreasing to 0·70 months) and did not have the same capacity for improvement.

Patterns of HIV testing after the scale-up of testing and treatment from 2010 onwards are presented in figure 1D, showing year-on-year increases in the annual number of tests, the proportion of tests among repeat testers (MSM with at least two tests within a 1-year period), and the overall number of repeat testers. This improved testing effort is set against a steady increase in the incidence of bacterial STIs from 2010 to 2018 (figure 1E), with gonorrhoea and syphilis diagnoses increasing at a rate of more than 2000 and 500 diagnoses per year, respectively. The plotted lines in figure 1E are scaled to show the increases in diagnoses of the two STIs relative to 2012 numbers across age groups. Both infections show the greatest increase in MSM aged 45 years or older. Figure 1F illustrates the scale of the PrEP phase-in between 2010 and 2018.

**Figure 2:**
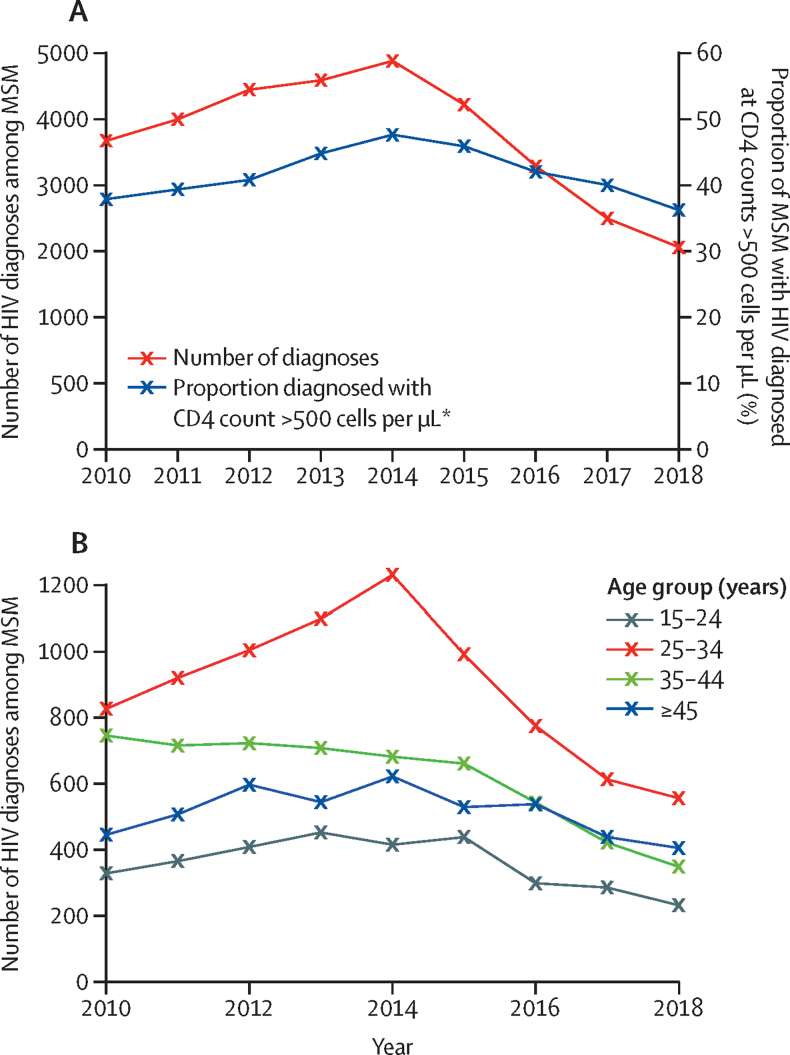
Annual number of new HIV diagnoses among MSM (A) Total annual number of HIV diagnoses, and percentage of HIV diagnoses with accompanying CD4 blood cells count within 3 months of diagnosis, among MSM in England. (B) Annual number of HIV diagnoses by age group. MSM=men who have sex with men. *CD4 count >500 cells per μL selected to reflect the early stage of HIV infection.

The increase in STI diagnoses during 2010–18 is in contrast to the trends in the number of new HIV diagnoses, which rose to a peak in 2014 and then markedly decreased (figure 2A). Stratification by age group (figure 2B) reveals a modest but sustained decline in HIV diagnoses throughout 2010–18 in MSM aged 35–44 years, contrasting with the sharp decrease in 2014 among those aged 25–34 years. After 2014, a modest decrease in diagnoses also became evident in MSM aged 15–24 years and eventually in those aged 45 years or older.

**Figure 3:**
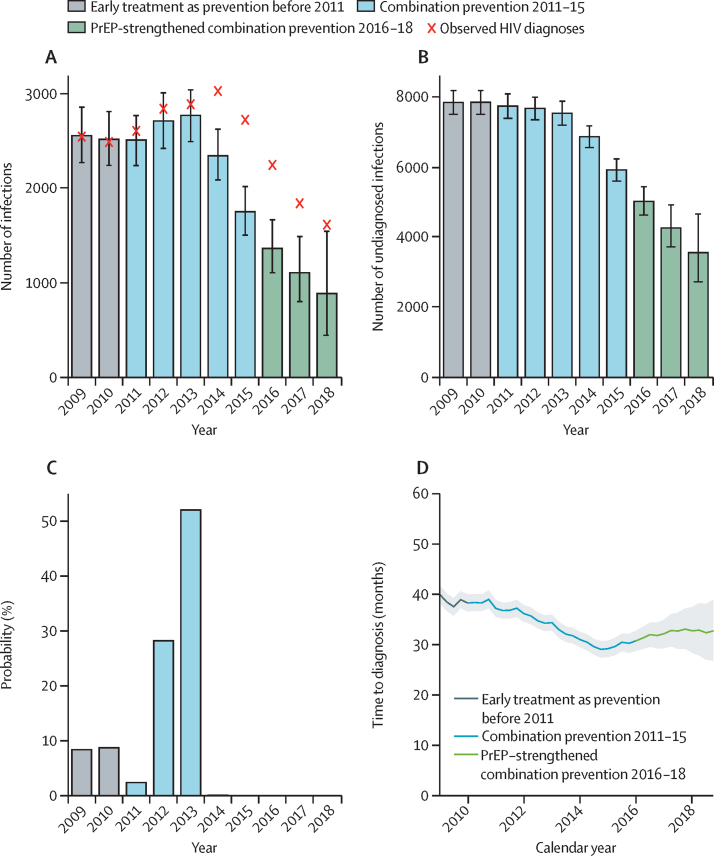
Back-calculation results (A) Estimated total annual number of new HIV infections (bars) against observed number of HIV diagnoses (crosses) among MSM in England. Error bars denote 95% CrIs. (B) Estimated number of undiagnosed infections in MSM by year (with 95% CrIs). (C) Distribution of the estimated peak year of new HIV infections. (D) Estimated mean time interval between HIV infection and diagnosis (solid line), measured in months, with 95% CrIs (shaded areas). PrEP=pre-exposure prophylaxis. MSM=men who have sex with men. CrI=credible interval.

Results from the model indicate a steep fall in the annual number of new infections from 2770 (95% credible interval [CrI] 2490–3040) in 2013 to 1740 (1500–2010) in 2015 (figure 3A). In the PrEP-strengthened era the decrease is sustained, with 854 (441–1540) new infections in 2018. The timing of the peak in infections (figure 3C) is estimated with approximately 80% certainty to be in 2012 or 2013, with 2013 being the most probable peak year (around 52% certainty). The estimated number of MSM living with undiagnosed HIV infection shows a gradual decline at the start of the intensified combination prevention era (figure 3B), with 7880 (95% CrI 7540–8220) undiagnosed infections at the end of 2010 decreasing to 7700 (7390–8040) by the end of 2013. This trend is followed by an accelerated fall in infections to 5930 (5610–6250) at the end of 2015, reaching a low of 3530 (2730–4670) by the end of 2018, representing a decrease of 40·5% in the 3 years after 2015.

The estimated mean time between infection and diagnosis decreased from 40·0 months (95% CrI 38·3–41·7) in 2009 to 29·0 months (27·4–30·6) in 2014, before increasing marginally to 32·7 months (26·8–38·9) in 2018 (figure 3D). The uncertainty associated with the estimated recent increase is large, and the increase is likely to be linked to the decreasing proportion of infections diagnosed at a CD4 count of more than 500 cells per μL (figure 2A).

**Figure 4:**
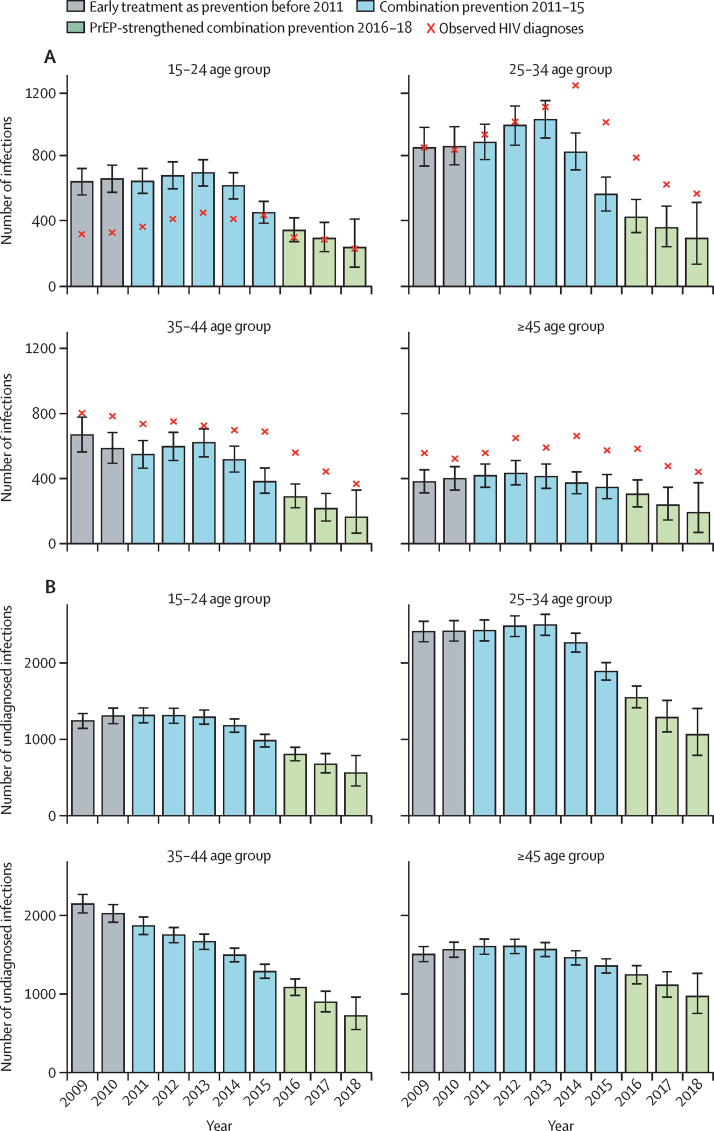
Back-calculation results stratified by age group Estimated annual number of new HIV infections (A) and undiagnosed infections (B), with associated 95% credible intervals. Crosses in (A) represent the observed annual number of new diagnoses.

When examined by age group, estimated trends in the number of new HIV infections shows some age heterogeneity. Infections in MSM aged 15–34 years (figure 4A) show a pattern consistent with the trend in all MSM (figure 3A), with steep decreases after 2013. In the 35–44 years age group, the number of new infections oscillates before 2013, after which a similar decrease to that in overall MSM is estimated. In MSM aged 45 years or older, the decrease occurred after 2012 and was more gradual than in other age groups. Age heterogeneity is also observed in the estimated number of undiagnosed infections, with MSM aged 15–34 years showing a decrease similar to the trend in new infections in this group. Undiagnosed infections in MSM aged 35–44 years are estimated to have steadily decreased throughout 2009–18. Trends in MSM aged 45 years or older are again more gradual than in other age groups, such that in this group the number of MSM living with undiagnosed HIV in 2018 was higher than in the 35–44 group (figure 4B).

**Figure 5:**
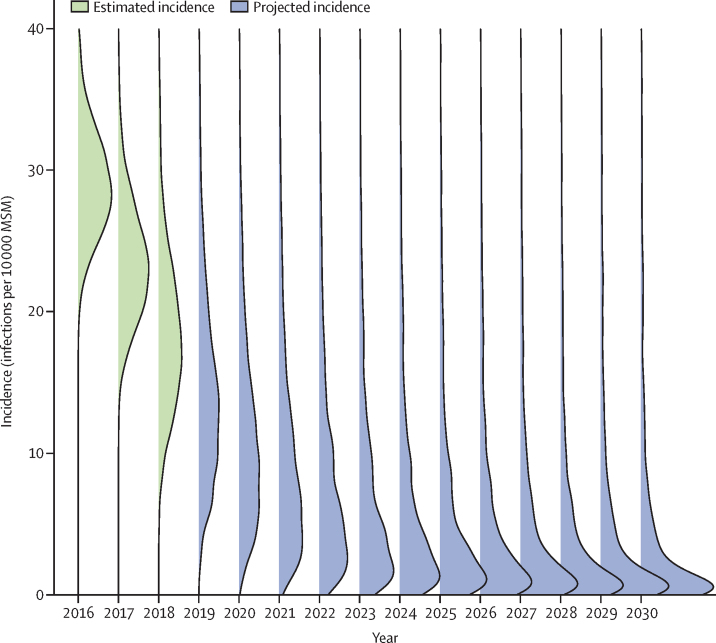
HIV incidence extrapolations Density plots of estimated incidence among MSM in 2016–18, with corresponding distributions for the projected annual incidence during 2019–30. MSM=men who have sex with men.

The MPES estimates showed that the number of HIV-negative MSM in England increased from around 456 000 in 2012 to 473 000 in 2018.^31^ Using the estimated annual number of new infections as numerator, we estimate an overall decrease in HIV incidence from 59·3 infections per 10 000 (95% CrI 49·6–71·0) to 37·5 infections per 10 000 (30·4–46·2) during the 2012–15 period, with a further decrease to 18·0 infections per 10 000 (9·20–33·0) by the end of 2018. Similarly, using the MPES estimates, we estimate a decline in undiagnosed prevalence during 2012–15 among all adult MSM, from 1·70% (95% CrI 1·46–1·98) to 1·28% (1·10–1·49), with a further decrease to 0·74% (0·56–1·03) by the end of 2018. Using the fitted back-calculation model for the number of new infections with a conservative assumption that the MSM denominator did not change from 2018 onwards, we extrapolated incidence annually up to 2030. We anticipate incidence to fall to five infections per 10 000 within 5 years, with an estimated mean incidence of 5·36 infections per 10 000 (95% CrI 0·398–56·3) in 2023, and 1·70 infections per 10 000 (0·019–110) in 2030 (figure 5). Despite the forecasts becoming increasingly uncertain, around 40% of the extrapolated incidence profiles were lower than the 1 infection per 10 000 level by 2030.

## Discussion

Our back-calculation analysis of the rich array of HIV surveillance information available in England has enabled estimation of age-specific trends in HIV incidence, prevalence of undiagnosed HIV infection, and the interval between HIV infection and diagnosis in MSM during the period 2009–18. The reconstructed HIV incidence showed the epidemic to be waning rapidly, despite large increases in the incidence of bacterial STIs, with a sharp downturn in the number of new HIV infections before the scale-up of PrEP, corresponding to the intensification of treatment as prevention policies in 2011–15. This decline was heterogeneous across adult age groups, being particularly rapid in MSM aged 25–34 years, and slowest in those aged 45 years or older. Furthermore, we examined HIV incidence extrapolations relative to target future dates for elimination of HIV transmission, and showed that elimination of HIV transmission as a public health issue might be within reach by 2030.

The observed decrease in the number of new HIV diagnoses since around 2014–15 has previously been correlated with the expansion in access to PrEP for MSM.^32^ However, we estimated the peak in number of new infections to have occurred during 2012–13, preceding the peak observed in HIV diagnoses by at least 18 months. Importantly, the decline in new infections began at least 1 year before the major scale-up of PrEP access from late 2015, and was most likely due to a continued expansion, during 2011–15, of HIV testing, together with more prompt treatment initiation following HIV diagnosis.

HIV incidence continued to decrease between 2016 and 2018, at a similar rate to that observed during 2014–15. The effect of further expansion of PrEP use during 2018, although a probable contributor to the sustained decline, is difficult to isolate from the continued increases in testing and immediate treatment.^33^ Nevertheless, the extra impetus of the introduction of a national PrEP programme in 2020 might cause an acceleration in the decrease in incidence, or at least ensure the decrease is maintained at its recent rate.

The slower decrease in HIV incidence for MSM aged 45 years or older could be because, during the period of study, this age group had the greatest proportional increase in new cases of syphilis and gonorrhoea, suggesting behavioural changes associated with increased risk of HIV transmission. Additionally, the introduction of immediate ART treatment for new HIV diagnoses was least likely to benefit an age group already experiencing a short time from HIV diagnosis to treatment initiation. In future, focused efforts to increase the number of regular HIV testers might benefit this age group in particular.

When compared with most high-income countries, the decrease in HIV incidence in England is noteworthy. For instance, HIV incidence in MSM has been estimated to have remained constant in the USA (50 infections per 10 000 MSM) and Australia (80 infections per 10 000 MSM) during 2012–16 and 2008–15.^34^, ^35^ In Canada, a small decrease from 50 infections per 10 000 MSM to 40 infections per 10 000 MSM between 2005 and 2014 has been estimated, with a stabilisation of incidence towards the end of 2016.^36^

CD4-staged back-calculation models do have limitations. Despite consistent increases in HIV testing, a commensurate fall in estimated time to diagnosis in recent years was not observed. One explanation for this is the assumption that all CD4 counts at seroconversion are high (>500 cells per μL), ignoring the short acute infection period when CD4 counts can rapidly decrease. Diagnoses in this period might be misclassified by the model as long-standing infections. This misclassification could be reduced by incorporation of information from serological tests for recent infection and dates of last negative tests, when available. These additional data would also add the benefit of more accurate estimates of incidence in the most recent years and reduced uncertainty in the forecasts. Such improvements are the focus of ongoing research.

A further limitation is that the model is not able to account for migration. MSM acquiring HIV outside of the UK might therefore have been classified as incident cases, although similar declines in diagnoses after 2014 for both UK-born and non-UK-born MSM indicate the majority of incidence occurring within the UK.^28^ Furthermore, in-country migration, in terms of movement between regions or changes in residence, could not be characterised. This inability to account for migration limits our ability to ascribe the decrease in incidence to a particular region, although epidemiological studies suggest that much of the early reduction in transmission occurred in London.^22^

Finally, the incidence extrapolations were obtained under the assumptions that testing, immediate treatment, and PrEP would be maintained at the most recent levels, and the consequent decrease in incidence would remain unchanged. According to our results, England could near the proposed elimination threshold of 1 new infection per 10 000 MSM per year by 2030. However, information systems monitoring each intervention show that their coverage could be further improved. If each intervention was maximised, the elimination rate might well be reached by 2030 or earlier. Even if adverse behavioural trends lead to a continued increase in bacterial STIs among MSM, optimising the biomedical HIV interventions might curtail future HIV transmission.

Although a direct causal link between the estimated HIV incidence trends and the different components of combination prevention cannot be established, this analysis provides an improved understanding of how prevention policies have shaped trends in HIV incidence, particularly where they have been differentially introduced and strengthened over time and across age groups. The lesson for other high-income countries from experience in England is that amplified testing and treatment as prevention have controlled the HIV epidemic in MSM at the country level. With additional large-scale implementation of PrEP, elimination of HIV transmission is likely to be within reach by 2030. However, targeted combination prevention measures might be needed to maintain the trajectory towards elimination in groups such as MSM aged 45 years or older. Additionally, to stay on track for elimination and ensure rapid and effective prevention policy adjustments, timely estimation of HIV incidence is essential, to recognise and respond appropriately to changes in the recent downward trend.

## Data sharing

The data used in this analysis are routine HIV and STI surveillance data curated by Public Health England and are available upon request. For further details about data availability and where to send a request please see the Public Health England HIV and STI data sharing policy.

## Declaration of interests

We declare no competing interests.
