# Supplementary material for: Tracking elimination of HIV transmission in men who have sex with men in England: a modelling study
Source: Lancet HIV. 2021 Jun 9;8(7):e440–8. doi: 10.1016/S2352-3018(21)00044-8 (PMC8238681; doi:10.1016/S2352-3018(21)00044-8)
Supplement: Supplementary appendix [file mmc1.pdf]

THE LANCET
HIV

Supplementary appendix

This appendix formed part of the original submission and has been peer reviewed. We post it as supplied by the authors.

Supplement to: Brizzi F, Birrell PJ, Kirwan P, et al. Tracking elimination of HIV transmission in men who have sex with men in England: a modelling study. *Lancet HIV* 2021; published online June 9. http://dx.doi.org/10.1016/S2352-3018(21)00044-8.

Appendix

The age and CD4-stage-specific back-calculation model

This model represents an extension of previous work, that used data on HIV diagnoses, information on the natural history of HIV and data on the disease stage at diagnosis to estimate the underlying, unobserved, HIV incidence in men-who-have-sex-with-men (MSM).[1] The extension consists of the specification of age at infection, so that age-time specific numbers of new HIV infections can be estimated, to enable a better characterisation of the HIV epidemic.

The model approximates the processes leading to HIV diagnosis through a multi-state structure that includes HIV infection, HIV progression and HIV diagnosis.

Figure 1 provides a schematic representation of the multi-state model. Here new HIV infections $h_{i,j}$, occurring in the ith time interval and j^{th} age group, progress through the $2K + 1$states: states $\{1, \ldots, K\}$ are undiagnosed states, describing disease progression through declining CD4-count; the remaining states represent AIDS diagnosis, and HIV diagnosis from the undiagnosed state is into states $\{K + 1, \ldots, 2K\}$.

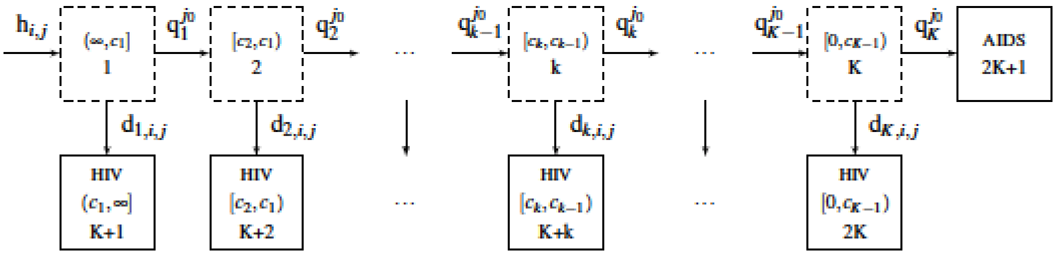

Figure 1. Schematic representation of the processes from HIV infection, progression and diagnosis (from Brizzi, 2018[2]).

Disease progression is specified by the progression probabilities $(q_1^{j_0}, q_2^{j_0}, \ldots, q_K^{j_0})$, which depend on the age at seroconversion (here denoted j_0), with individuals infected at an older age progressing more quickly (CASCADE Collaboration, 2000). The diagnosis probabilities $(d_{1,i,j}, \ldots, d_{K,i,j})$depend on (current) age and calendar time, to reflect that the propensity to test for HIV may vary with age and be affected by testing campaigns. Specifically, we assume K=4, with CD4 count categories defined as (∞,500], [350,500), [200,350), [0,200); and consider the [15-25), [25-35), [35,45), 45+ age groups.

Data on quarterly new HIV and AIDS diagnoses, i.e. on the number entering the AIDS and HIV diagnosis compartments, are available from the HIV and AIDS Reporting System (HARS)[3]. These, together with linked information on CD4 counts at the time of HIV diagnosis[4] and evidence from cohort studies on HIV progression (see below), are used to estimate the number of new infections and diagnosis probabilities.

To do so, the $h_{i,j}$, the (expected) number of new infections, is modelled flexibly as a smooth function through a thin-plate regression spline[5,] which allows the number of new infections to

vary smoothly across neighbouring ages and times. The diagnosis probabilities $d_{k,i,j}$ are also allowed to vary smoothly over time and age and are modelled using a random walk process on the logistic scale. Both models for the $h_{i,j}$ and $d_{k,i,j}$ are expressed in terms of parameters which are estimated.[2,6]

Disease progression

The age-specific disease progression probabilities have been separately estimated from the CASCADE cohort[7] and in this study are therefore assumed to be fixed and known. Summaries of these are given in the Table below.

Age at infection	State 1 (CD4 ≥ 500)	State 2 (500 < CD4 ≤ 350)	State 3 (350 < CD4 ≤ 200)	State 4 (CD4 > 200)	Time to AIDS
15-24	2.77	2.13	2.23	1.76	8.90
25-34	2.70	2.14	2.19	1.73	8.76
35-44	2.62	2.13	2.13	1.68	8.54
45-54	2.53	2.08	2.03	1.59	8.23
55-64	2.50	2.08	1.92	1.47	7.97
65+	2.30	1.86	1.68	1.31	7.16

Table S1: Mean time (in years) spent in each undiagnosed state, stratified by age at infection.

Extrapolation

Once the spline describing the number of new infections has been estimated, evaluation of the spline at future time-points allows the number of infections to be extrapolated into the future. In practice this involves a continuation of the linear trend in each age-group in the short-term, with each age-group gradually 'regressing to the mean' trend in the wider population in the longer term.[5]

Code to carry out this extrapolation is available in the gitlab repository associated with this work (https://gitlab.com/pjbirrell/agedep-backcalc-mcmc), within the file R/future_pred.R.

Limitations

- The structure of the model assumes a gradual decline in CD4 count over time from seroconversion to diagnosis. This ignores the initial phase of acute infection when there may be a sharp and transient drop in the CD4 count. This could potentially mean that some diagnoses in the acute phase are misclassified as long-standing infections, although historically the number of diagnoses occurring during seroconversion is thought to be low.

- The model assumes a closed sub-population of undiagnosed infections in England. Consequently, diagnoses are removed from the data if the individual was both born abroad and had an initial diagnosis abroad.
- The requirement for a closed population of undiagnosed infections limits the capacity for this model to stratify by region.
- Transmission risk is unknown in a small fraction of diagnoses among adult men. These diagnoses are randomly assigned to be in MSM assuming that the missingness is non-informative, given the age at diagnosis. From repeat running of the model, inferences appear robust to this imputation.
- The dataset is assumed to be complete after six months. Data were available for a further two quarters (six months) beyond the period modelled here, but these were omitted because of the likely presence of reporting delay. Reporting delays greater than six months are assumed to be rare, due to the linkage between HARS and HIV service commissioning, as discussed in the *Data Sources* section.

Additional references

1. P. J. Birrell, T. R. Chadborn, O. N. Gill, V. C. Delpech, D. De Angelis, Estimating Trends in Incidence Time-to-Diagnosis and Undiagnosed Prevalence using a CD4-based Bayesian Back-calculation, Stat Commun Infect Dis 4 (2012).
2. Brizzi F (2018) Estimating HIV incidence from multiple sources of data. PhD thesis, University of Cambridge
3. Rice BD, Yin Z, Brown AE et al. Monitoring of the HIV epidemic using routinely collected data: the case of the United Kingdom. AIDS Behav 21 (Suppl 1) (2017) 83–90.
4. Public Health England. National HIV surveillance data tables. Available from: https://www.gov.uk/government/statistics/hiv-annual-data-tables.
5. S. N. Wood, Thin plate regression splines, Journal of the Royal Statistical Society: Series B (Statistical Methodology) 65 (2003) 95-114.
6. F. Brizzi, P. J. Birrell, M. T. Plummer, et al., Extending Bayesian back-calculation to estimate age and time specific HIV incidence, Lifetime Data Anal. 25 (2019) 757-780.
7. Collaborative Group on AIDS Incubation and HIV Survival including the CASCADE EU Concerted Action. Time from HIV-1 seroconversion to AIDS and death before widespread use of highly-active antiretroviral therapy: a collaborative re-analysis. Lancet 2000; 355: 1131–37.
